# Addition of Trastuzumab Deruxtecan to Selpercatinib in a Patient With RET Fusion-Driven NSCLC and an Acquired HER2 Amplification: Case Report

**DOI:** 10.1016/j.jtocrr.2023.100603

**Published:** 2023-11-15

**Authors:** Chetan V. Vakkalagadda, Jyoti D. Patel

**Affiliations:** a Department of Medicine, Division of Hematology and Oncology, Northwestern University, Chicago, Illinois; b Knight Cancer Institute, Oregon Health and Science University, Portland, Oregon

**Keywords:** RET, Her2 amplification, Selpercatinib, Fam-trastuzumab deruxtecan, Next-generation sequencing, Case report

## Abstract

Despite the high activity of selective RET inhibitors in RET-driven NSCLC, resistance eventually develops and there is unmet need to better define therapeutic options for patients. This is a case of a patient initially thought to have no targetable alterations, then found to have a RET fusion, and subsequently HER2 amplification on three distinct biopsies. She was treated initially with chemotherapy and immune therapy, then switched to selpercatinib, and eventually had fam-trastuzumab deruxtecan added to selpercatinib. She also developed neuroendocrine differentiation at time of progression in the context of a p53 mutation, which is a known factor that can lead to small cell transformation. This patient’s case highlights the need for comprehensive molecular testing at both diagnosis and progression, as unexpected resistance mechanisms may be identified particularly for patients with uncommon driver mutations.

## Introduction

RET fusions are known driver mutations in advanced lung cancers, found in 1% to 2% of patients with NSCLC. Selpercatinib and pralsetinib are approved by the Food and Drug Administration (FDA) as first-line therapies. Data are emerging regarding mechanisms of resistance to RET therapies. A retrospective study of 18 patients treated with RET inhibitors identified RET resistance mutations (on-target), MET amplification, and KRAS amplification as the clearest mechanisms of resistance.^1^ Small cell transformation has also been reported in one patient treated with pralsetinib who had a P53 mutation in addition to a CCDC6:RET fusion.^2^ HER2 amplification has not been described in the literature as a known resistance mechanism in RET-driven NSCLC. Furthermore, although HER2-directed therapies have been FDA approved in HER2-mutated NSCLC, there are limited data on their efficacy in HER2-amplified NSCLC. We here present a case of a patient with NSCLC with RET fusion treated with selpercatinib who, at progression, was found to have HER2 amplification on tissue biopsy. The addition of fam-trastuzumab deruxtecan to selpercatinib maintained a response and was well tolerated with minimal side effects.

## Case Presentation

A 65-year-old woman with no history of tobacco use was diagnosed with having lung adenocarcinoma in September 2018. She was diagnosed at a local hospital after developing persistent cough. Chest computed tomography (CT) with contrast revealed a left upper lobe mass 3 cm in size with adjacent consolidation and lymphadenopathy. Bronchoscopic biopsy results of the left upper lobe lesion and lymph nodes level 7, 4R, and 10R were all consistent with pulmonary adenocarcinoma, CK7/TTF-1 positive, and homogenous expression of p53 on immunohistochemistry indicating a mutation, with programmed death-ligand 1 expression of 30%. No targetable mutations were found on a limited 22-gene targeted DNA cancer panel (Table 1). Staging positron emission tomography (PET)/CT confirmed avidity of the intrapulmonary findings with maximum standardized uptake value (SUVmax) of 11.9, a small left pleural effusion, and F-fluorodeoxyglucose–avid lesions in the liver and left ischium. Magnetic resonance imaging (MRI) result of the brain was negative for metastasis. Her initial TNM stage was T1c N3 cM1b.

**Table 1:** Date of Each Biopsy, Location, Histologic Diagnosis, Nature of NGS Testing Sent, and Identified Known and Potentially Relevant Mutations

Date	Site of biopsy	Biopsy Results	Type of NGS Panel Sent	Results—Known Clinical Significance	Results—Possible Clinical Significance
October 2018	Lung and mediastinal lymph nodes	Adenocarcinoma - TTF-1, CK7 positive - P40, WT1, PAX8 negative - PD-L1 30% - TP53 loss (IHC)	Internal 22-gene panel—local	Negative	Negative
May 2020	Left supraclavicular lymph node	Metastatic adenocarcinoma, favor lung primary - TTF-1, AE1/AE3 positive - Napsin A, GATA3 negative - PD-L1 5%	Tempus comprehensive panel	KIF5B-RET fusion	SETD2 p.(Q960∗) TP53 p.(C238F) ARID1A loss of function CDKN2A loss of function CDKN2B loss of function FGFR3 amplification PTEN loss of function
May 2022	Left cervical lymph node	Metastatic poorly differentiated carcinoma with neuroendocrine features, consistent with lung primary - TTF-1, CD56, CK7 positive - P40, synaptophysin, chromogranin, CK20, CD45, NSE negative - Ki-67 80%–90%	Oncomine Precision (OPA) NGS panel—internal	KIF5B-RET fusion ERBB2 amplification IDH2 p.(R172M)	IDH2 TP53 p.(C238F) TP53 p.(R273H) FGFR3 amplification PIK3CA amplification PTEN loss

IHC, immunohistochemistry; NGS, next-generation sequencing; PD-L1, programmed death-ligand 1.

**Figure 1:**
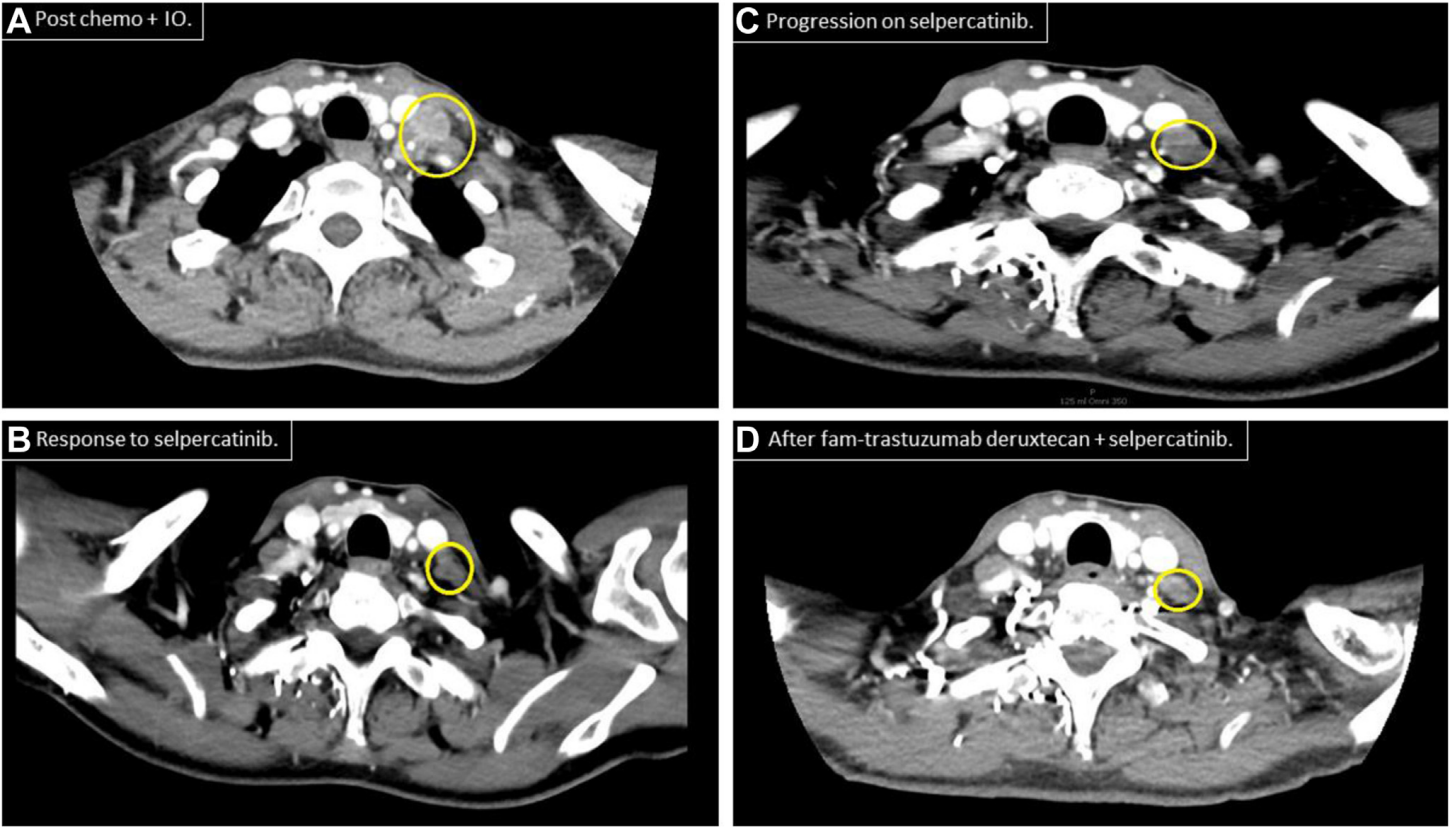
CT scans of the neck with the relevant lymph nodes circled. Panel *A* was before initiation of selpercatinib (1.6 cm in size), panel *B* with decrease in size after 15 months of selpercatinib, panel *C* at time of progression in the neck, and panel *D* after SBRT and then eight cycles of fam-trastuzumab deruxtecan plus selpercatinib. Chemo, chemotherapy; CT, computed tomography; IO, immune-oncology; SBRT, stereotactic body radiation therapy.

She first underwent local control with radiation therapy to the left upper lobe and left hilum at 35 Gy in 14 fractions. She then began treatment with carboplatin area under the curve 5, pemetrexed 200 mg/m^2^, and pembrolizumab 200 mg every 3 weeks for four cycles, followed by maintenance pemetrexed and pembrolizumab, achieving a sustained partial response. After 15 months, PET scan in February 2020 revealed a new 8-mm left supraclavicular lymph node with SUVmax of 3.5. Interval CT scan of the neck in May 2020 revealed further increase in size to 1.6 cm (Fig. 1*A*). Lymph node biopsy result returned consistent with adenocarcinoma of pulmonary origin with programmed death-ligand 1 score of 5%. Both Tempus comprehensive next-generation sequencing (NGS) tissue testing and Guardant360 circulating tumor DNA revealed a KIF5-RET fusion (Table 1).

Given oligoprogression, she received 20 Gy in five fractions to the left supraclavicular node and continued treatment with maintenance pemetrexed and pembrolizumab until September 2020, when MRI result of the brain revealed a new enhancing central nervous system lesion. At this time, she also developed grade 2 arthralgias and grade 2 hepatitis related to immunotherapy. Thus, pemetrexed plus pembrolizumab was discontinued, and 1 month after, she began treatment with selpercatinib 160 mg twice a day. She had no further episodes of hepatitis, and the lesion found on MRI on the brain resolved without other intervention. CT imaging initially showed response to selpercatinib (Fig. 1*B*).

After 17 months on selpercatinib, she developed recurrent enlargement of a left cervical lymph node in an adjacent area to the prior area of progression (Fig. 1*C*). PET/CT results revealed uptake in this lymph node, right adrenal gland (SUVmax 5.7) and T10 vertebral body (SUVmax 3.6). Result of a core biopsy of the left cervical lymph node revealed poorly differentiated carcinoma, positive for TTF-1, CD56, and cytokeratin 7; Ki-67 of 80% to 90%; and negative for all other markers. The pathology result was consistent with lung primary and neuroendocrine features. NGS result revealed persistence of KIF5B-RET fusion and now ERBB2 (HER2) amplification with gene copy number of 10.6 (Table 1).

**Figure 2:**
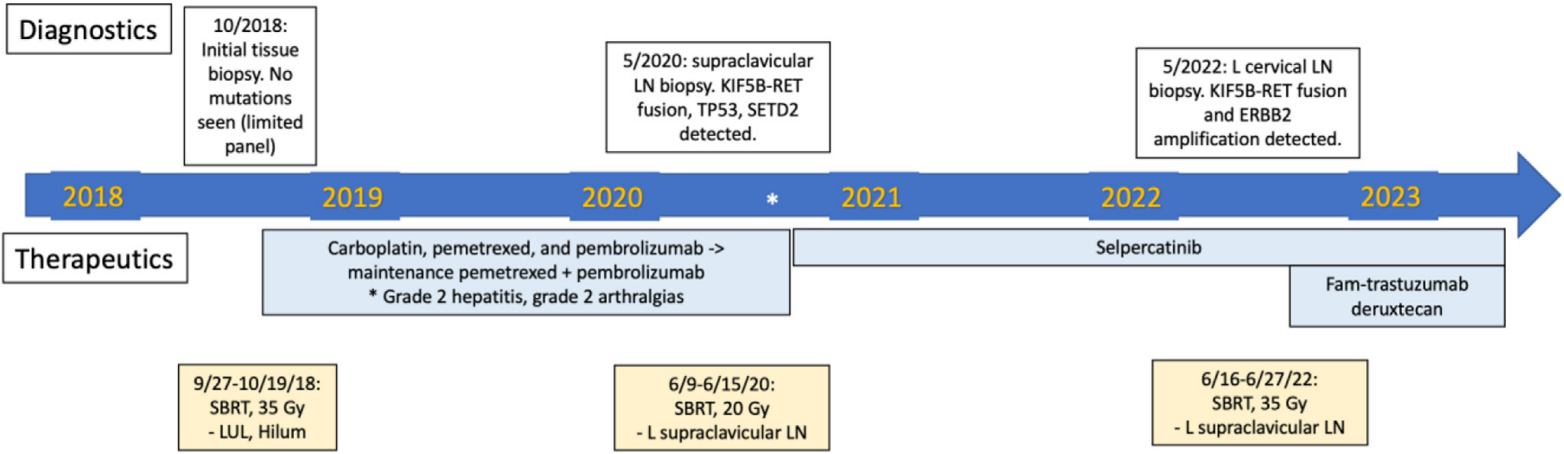
Timeline of events from diagnosis to present. Diagnostics (biopsies) are on top, and therapeutics (medications, radiation therapy) are on bottom. LN, lymph node; SBRT, stereotactic body radiation therapy.

She continued selpercatinib, and fam-trastuzumab deruxtecan was initiated (5.4 mg/kg every 21 d). CT scans (Fig. 1*D*), PET scans, and MRI scans of the brain have since revealed response in the adrenal gland and vertebral body. She has tolerated treatment well experiencing grade 1 fatigue and neuropathy. A timeline of her diagnostic and treatment course is shown in Figure 2.

## Discussion

This is the first reported case of acquired ERBB2/HER2 amplification as a resistance mechanism to RET inhibition in advanced NSCLC. In this case, our patient also developed neuroendocrine features while on therapy, potentially in the context of a concurrent p53 mutation. This case highlights the importance of comprehensive molecular profiling of NSCLCs on both tissue and blood, as the RET fusion was identified 2 years after diagnosis as the first panel was limited.

HER2 amplifications are found in 2% to 5% of patients with NSCLC and are one of three HER2 alterations observed in patients with lung cancer, the other two being HER2 mutations (1%–4%) and HER2 overexpression (2%–30%).^3^ HER2 amplifications can be identified by fluorescence in situ hybridization (evaluating the ratio of the HER2 gene copy number to the chromosome enumeration probe 17, and positive if the ratio is greater than 2), NGS (positive if copy number is greater than 6), enzyme-linked immunosorbent assay, or reverse-transcriptase polymerase chain reaction.^3^ HER2 amplifications and overexpression can co-exist with HER2 mutations and can also develop as mechanisms of resistance to other targeted therapies.

Trastuzumab deruxtecan is FDA approved as a second-line option for patients with HER2 mutations, but not overexpression or amplification. This was based on the DESTINY-Lung01 trial, in which 91 patients with previously treated HER2-mutated advanced NSCLC received trastuzumab deruxtecan and among whom a 55% response rate was found with median progression-free survival of 8.2 months and median overall survival of 17.8 months.^4^ Two of 91 patients also had HER2 amplifications, and both had a deep response. A separate subgroup analysis of DESTINY-Lung01 evaluated trastuzumab deruxtecan in HER2-overexpressing, previously pretreated NSCLC, at a dose of 6.4 mg/kg every 3 weeks. Among 49 patients, objective response rate was 24.5%, median depth of response 6.0 months, disease control rate 69.4%, and median progression-free survival 5.4 months.^5^ Data are limited regarding response of HER2-amplified tumors to targeted agents. A phase 2 basket trial of ado-trastuzumab emtansine in multiple HER2-amplified cancers revealed an objective response rate of 26% with three of six patients with HER2-amplified NSCLC achieving a response.^6^ The depth of response to trastuzumab deruxtecan in this patient underscores the need for further work in understanding response of HER2-amplified lung tumors to anti-HER2 therapy.

More broadly, the change in mutations at each progression indicates the importance of comprehensive molecular testing. As more patients are treated with targeted therapies and comprehensive testing becomes more widespread, the incidence of atypical resistance mechanisms or co-mutations is likewise increasing. Novel combinations of therapy will likely be needed for more patients as we continue to understand the changing molecular signatures of oncogene-driven NSCLC in the entire time course of treatment.

## Conclusion

HER2 amplifications and neuroendocrine changes can both be found in patients with RET fusion-driven NSCLC, as reported in this case. Concomitant p53 mutations with RET fusions can indicate higher risk for neuroendocrine transformation. The combination of fam-trastuzumab deruxtecan and selpercatinib with RET fusion and HER2 amplification is an effective and safe combination therapy.

## CRediT Authorship Contribution Statement

**Chetan V. Vakkalagadda:** Data curation, Investigation, Formal analysis, Writing—original draft, Writing—review and editing, Visualization.

**Jyoti D. Patel:** Conceptualization, Writing—review and editing, Supervision, Project administration.

## Informed Consent

Consent was obtained from the patient to publish her case in the medical literature.
